# O Efeito da Administração Precoce de Solução Salina Hipertônica na Insuficiência Cardíaca Descompensada Aguda

**DOI:** 10.36660/abc.20230818

**Published:** 2024-07-01

**Authors:** Tugce Colluoglu, Tuğba Kapanşahin, Melahat Hicran Aksu, Orhan Önalan, Yeşim Akin

**Affiliations:** 1 Karabuk University Faculty of Medicine Department of Cardiology Karabuk Turquia Karabuk University, Faculty of Medicine, Department of Cardiology, Karabuk – Turquia

**Keywords:** Solução Salina Hipertônica, Testes de Função Renal, Insuficiência Cardíaca

## Abstract

**Fundamento:**

Não houve evidência científica sobre o tratamento inicial com solução salina hipertônica (SSH) na insuficiência cardíaca agudamente descompensada (ICAD).

**Objetivos:**

Este estudo avaliou o impacto do uso de SSH junto com um diurético de alça (DA) como o primeiro tratamento diurético para ICAD, com foco na função renal, níveis de eletrólitos e resultados clínicos.

**Métodos:**

Neste estudo retrospectivo de caso-controle, 171 pacientes adultos (93 mulheres/78 homens) com ICAD foram incluídos entre 1º de janeiro de 2022 e 31 de dezembro de 2022. Os pacientes foram alocados em dois grupos: combinação inicial de SSH+DA e DA padronizada. O desfecho primário foi piora da função renal (PFR). A hospitalização por IC e a mortalidade por todas as causas foram avaliadas durante 6 meses de acompanhamento. O nível de significância adotado na análise estatística foi de 5%.

**Resultados:**

Os grupos exibiram semelhanças nas características basais. Diurese significativamente maior no 1º dia (3975 [3000-5150] vs. 2583 [2000-3250], p=0,001) e natriurese na 2ª hora (116,00 [82,75-126,00] vs. 131,75-140,00] vs. 94,00-103,25] vs. 99,00 [96,00-103,00], p=0,295), TFG (48,50 [29,75-72,50 vs. 50,00[35,50-63,50, p=0,616) e creatinina (1,20 [0,90-1,70] vs. 1,20 [1,00-1,50], p=0,218) permaneceu estável no grupo SSH combinado inicial quando comparado ao grupo DA padronizado (Cl-: 102,00[99,00-106,00] vs. 98,00[95,00-103,00], p=0,001, TFGe: 56,00 [41,00-71,00] vs. 55,00[35,00-71,00], p=0,050, creatinina: 1,10[0,90-1,40] vs. 1,20 [0,90-1,70], p=0,009). A piora da função renal (16,1% vs. 35,5%, p = 0,007) e o tempo de internação hospitalar (4 dias [3-7] vs. 5 dias [4-7], p = 0,004) foram menores na combinação inicial SSH+DA em comparação com o DA padronizado. A mortalidade hospitalar, a hospitalização por IC e a mortalidade por todas as causas foram semelhantes entre os dois grupos.

**Conclusão:**

SSH como terapia inicial, quando combinada com DA, pode proporcionar uma diurese segura e eficaz sem prejudicar a função renal na ICAD. Portanto, a SSH pode levar a um menor tempo de internação hospitalar para esses pacientes.

## Graphical abstract

**Figura Central:**
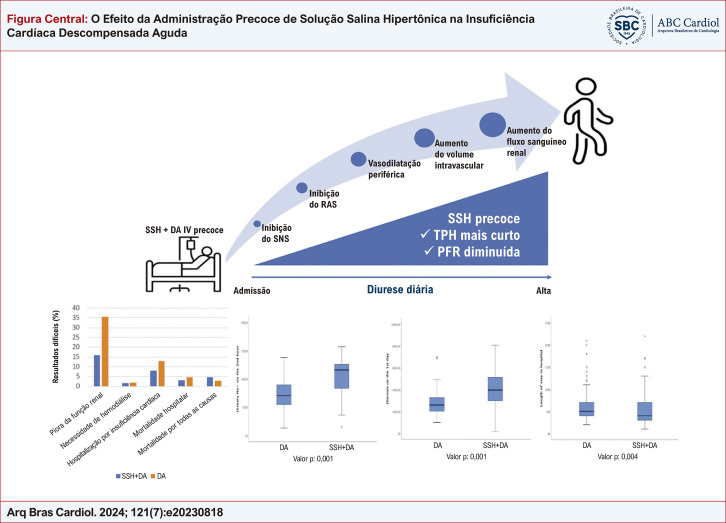
: O Efeito da Administração Precoce de Solução Salina Hipertônica na Insuficiência Cardíaca Descompensada Aguda

*SSH: solução salina hipertônica DA: Diuréticos de alça SNS: sistema nervoso simpático SRAA: sistema renina-angiotensina-aldosterona TPH: Tempo de permanência no hospital PFR: Piora da função renal.*

## Introdução

O agravamento da insuficiência cardíaca (AIC) está associado ao aumento da mortalidade e morbilidade, contribuindo para uma carga económica significativa para a sociedade. Esses episódios são o principal fator por trás da hospitalização por insuficiência cardíaca (IC), principalmente devido ao agravamento da congestão que requer diuréticos intravenosos (IV).^1^ As diretrizes atuais para IC recomendam um regime diurético escalonado que inclui a administração de IV diuréticos de alça como terapia de primeira linha para agravamento da congestão. No entanto, com IV apenas com diuréticos de alça, o tempo necessário para atingir a descongestão ideal é estendido, resultando em muitos pacientes com IC recebendo alta com congestão residual. A presença de congestão residual está ligada a desfechos desfavoráveis e readmissão precoce.^2,3^ É digno de nota que os diuréticos do túbulo proximal estão sendo testados para melhorar a terapia descongestiva além do tratamento padrão baseado em diuréticos de alça, incluindo acetazolamida e inibidores do cotransportador 2 de sódio-glicose.^3-6^

Na literatura, NaCI oral ou solução salina hipertônica (SSH) IV de NaCI a 3% tem sido usada para melhorar a resposta diurética em pacientes com IC refratária e agudamente descompensada com perfil seguro. A produção de urina e a perda de peso aumentaram significativamente com a administração de SSH e altas doses IV De diurético de alça nesta população.^7-9^ No entanto, os estudos acima mencionados avaliaram o efeito do SSH como uma opção de resgate entre pacientes com IC refratária e agudamente descompensada.^7,9-11^ O efeito da administração inicial de SSH como parte de um regime diurético inicial tem sido pouco estudado na IC agudamente descompensada.^12^ À medida que examinamos o corpo de literatura existente, torna-se evidente que existe uma lacuna na investigação do benefício potencial da administração proactiva de SSH, em oposição à sua utilização convencional como intervenção de resgate, particularmente após a publicação de ensaios significativos que ligaram o seu uso com um regime diurético combinado como terapia inicial na insuficiência cardíaca agudamente descompensada (ICAD). Portanto, pretendemos avaliar se a adição inicial de SSH à terapia padronizada com diurético de alça IV entre pacientes com IC agudamente descompensada melhoraria a resposta diurética, o descongestionamento bem-sucedido, o tempo de internação hospitalar e desfechos difíceis com segurança.

### Desenho do estudo e população do estudo

Este estudo foi conduzido como um estudo retrospectivo de caso-controle. O protocolo do estudo foi aprovado e conduzido seguindo o número de aprovação 2023/1294 do Comitê de Ética para Pesquisa Clínica Não Intervencionista da Universidade Karabuk. As Diretrizes STROBE para estudos de caso-controle e uma lista de verificação foram utilizadas na preparação deste manuscrito.^13^ O estudo foi realizado de acordo com os princípios estabelecidos na Declaração de Helsinque.

### Cálculo do tamanho da amostra

De acordo com a Fórmula Kelsey, 111 (43/68) pacientes são necessários para uma potência de 80%, e determinamos uma redução de risco relativo de 70% no desfecho primário com a terapia com SSH com base no ensaio de Issa et al.^12,14^

### Coleção de dados

**Figura 1:**
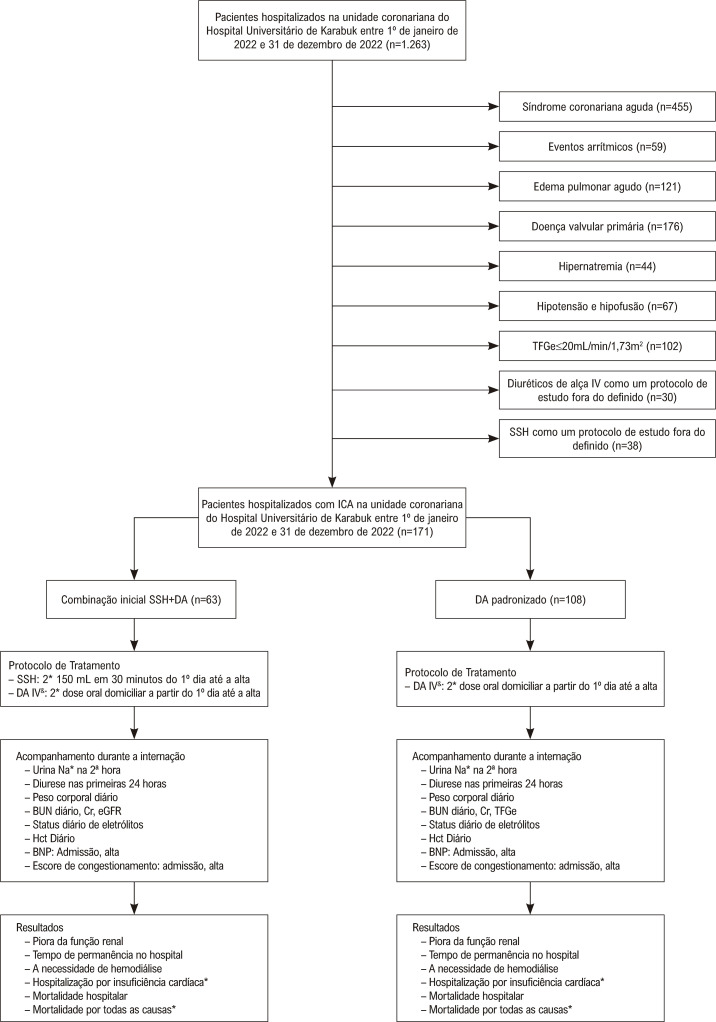
– Fluxograma. &: Em casos de resposta inadequada a diuréticos de alça IV, a dose de diuréticos de alça IV é aumentada duas vezes. *: A hospitalização por insuficiência cardíaca e a mortalidade por todas as causas foram avaliadas nos primeiros 6 meses após a hospitalização índice. TFGe: taxa de filtração glomerular estimada; IV: intravenoso; SSH: solução salina hipertônica; ICA: insuficiência cardíaca agudamente descompensada; DA: diurético de alça; BUN: nitrogênio na urina no sangue; Cr: creatinina; BNP: peptídeo natriurético cerebral; Htc: hematócrito.

Identificamos todos os pacientes em toda a faixa de fração de ejeção do ventrículo esquerdo (FEVE) hospitalizados com diversas causas cardíacas entre 1º de janeiro de 2022 e 31 de dezembro de 2022, na unidade de terapia intensiva coronariana do Hospital Universitário de Karabuk, a partir dos registros médicos eletrônicos do hospital (n=1263). Os critérios de inclusão foram evidências de IC agudamente descompensada, confirmada pelos níveis elevados de peptídeo natriurético tipo B (BNP) (≥100 pg/mL), achados de congestão na ecocardiografia e radiografia de tórax ou tomografia computadorizada de tórax. Pacientes que receberam SSH como tratamento de resgate que não o receberam no primeiro dia (n=38), pacientes que receberam tratamento IV com diuréticos de alça como um protocolo de estudo fora do definido (n=30), taxa de filtração glomerular estimada (TFGe) ≤20 mL/min/1,73m^2^ (n=102), pressão arterial sistólica ≤90 mmHg (n=43) ou sinais de hipoperfusão definida pelos níveis de lactato ≥2mmol/L (n=24), hipernatremia (Na+ ≥145mEq/L) (n=44), edema pulmonar agudo (n=121), síndrome coronariana aguda (n=455), eventos arrítmicos (n=59) e doença valvular primária (n=176) não foram incluídos. Portanto, este estudo incluiu 171 pacientes com IC agudamente descompensada. O grupo controle consistiu em 108 pacientes escolhidos consecutivamente, de mesma idade e sexo, O grupo de controle consistiu em 108 pacientes escolhidos consecutivamente, de mesma idade e sexo, cujos diuréticos foram administrados apenas como diuréticos de alça IV em bolus (Figura 1).

Variáveis como idade, sexo, comorbidades, terapia médica prescrita por diretrizes para insuficiência cardíaca, diuréticos de alça prescritos, data de internação, tempo de permanência no hospital, estado de hemodiálise e data do óbito foram relatadas. Variáveis laboratoriais, incluindo testes de função renal (nitrogênio na urina, creatinina [Cr], TFGe), BNP, estado eletrolítico (Na+, K+, Cl-, Mg++, Ca++), Na+ urinário na 2ª hora, hemoglobina e hematócrito (Htc), foram obtidos do banco de dados de prontuários eletrônicos do hospital. O peso corporal e o débito urinário diário foram obtidos a partir do relatório do exame físico diário. A doença renal crônica (DRC) foi definida como uma taxa de filtração glomerular estimada (TFGe) de <60 mL/min/1,73m^2^ calculada por pelo menos 3 meses.^15^ O tempo de permanência no hospital foi obtido no banco de dados eletrônico de saúde do hospital. Os dados sobre mortes foram obtidos do Sistema Turco de Notificação de Óbitos.

### Protocolo de solução salina diurética e hipertônica

Os diuréticos de alça intravenosos foram administrados com o dobro da dose dos diuréticos domiciliares, de acordo com a Diretriz 2022 da AHA/ACC/HFSA para o Tratamento da Insuficiência Cardíaca.^16^ O grupo caso foi composto por 63 pacientes que receberam 150 mL de SSH durante 30 minutos (300 mL/h) duas vezes ao dia, desde o primeiro dia de internação até a alta.^7^

### A medição da fração de ejeção do ventrículo esquerdo

Todos os exames ecocardiográficos foram realizados com o Philips EPIQ-7C Ultrasound System for Cardiology (Andover, EUA) com sonda X5-1 tanto na admissão quanto na alta. Todos os dados ecocardiográficos foram armazenados para análise offline. A FEVE foi medida pelo método de Simpson biplano modificado.

### A definição de resultados e parâmetros de segurança

O desfecho primário foi a piora da função renal, que foi definida como um aumento no nível de creatinina plasmática de ≥0,3 mg/dL durante a estratégia de tratamento diurético.^17^ Os desfechos secundários foram a necessidade de hemodiálise, descongestão bem-sucedida, tempo de internação hospitalar, hospitalização por IC e mortalidade por todas as causas. O débito urinário foi definido como a resposta à estratégia diurética durante as primeiras 24 horas. O congestionamento foi avaliado duas vezes: no momento da admissão e no momento da alta hospitalar. Realizamos uma avaliação abrangente para avaliar a congestão pulmonar e sistêmica. O escore de congestão foi estabelecido por meio do uso de documentação médica e de um sistema de escore simples que atribuiu um ponto para cada achado de congestão (Tabela Suplementar 1). O tempo de internação foi definido como o período entre a data da admissão hospitalar e a data da alta hospitalar. A hospitalização por IC e a mortalidade por todas as causas foram avaliadas nos primeiros 6 meses após a data da alta índice.

### Análise estatística

A normalidade das variáveis contínuas foi avaliada através do teste de Kolmogorov-Smirnov. Variáveis contínuas foram expressas como média±desvio padrão ou mediana (intervalo interquartil), conforme apropriado. As variáveis categóricas foram expressas em números e porcentagens. O teste Qui-Quadrado, para variáveis categóricas, e o teste t de Student não pareado ou o teste U de Mann-Whitney, para variáveis contínuas, foram realizados para comparar as características basais e os efeitos do tratamento entre duas estratégias de tratamento, conforme apropriado. Para avaliar as diferenças nos parâmetros laboratoriais e no peso corporal antes e depois de duas estratégias de tratamento distintas, foi realizado o teste de Wilcoxon. O tamanho do efeito entre as duas estratégias de tratamento foi analisado pelo tamanho do efeito r de Cohen: z/√n. A definição do tamanho do efeito, calculado pelo coeficiente r, é: r=0,10 é considerado um efeito pequeno, r=0,30 é um efeito médio e r=0,50 é um efeito grande.^18^ Um valor p bicaudal de 0,05 foi considerado estatisticamente significativo. Todas as análises foram realizadas no software estatístico SPSS versão 25.0.

## Resultados

### Características base

As características basais de 171 pacientes estão resumidas na Tabela 1. A idade média dos pacientes foi de 73,7±10,1 anos e 93 (54%) dos pacientes eram mulheres. Os pacientes com IC foram classificados em dois grupos com base na abordagem inicial do tratamento diurético (Tabela 1).

**Tabela 1:** – Características basais da população estudada

	SSH+LD Combinação inicial (n = 63)	LD Padronizado (n = 108)	p
**Idade (anos)**	74,3± 9,2	73,3±10,7	0,557
**Sexo (F/M)**	36/27	57/51	0,580
**FEVE (%)**	45.00 (35.00-55.00)	45.00 (35.00-59.00)	0,414
**Comorbidades**			
Hipertensão (n,%)	50 (79,4)	92 (85,2)	0,328
Diabetes Mellitus (n,%)	35 (55,6)	57 (52,8)	0,725
CAD (n,%)	27 (42,9)	52 (48,1)	0,503
Hiperlipidemia (n,%)	25 (39,7)	46 (42,6)	0,710
DRC (n,%)	45 (76,3)	77 (68,8)	0,301
Fibrilação Atrial (n,%)	31 (50,0)	50 (46,7)	0,682
**Terapia Médica Básica**			
Betabloqueadores (n,%)	60 (96,8)	101 (94,4)	0,482
RASi (n,%)	40 (64,5)	79 (73,8)	0,201
ARM (n,%)	45 (72,6)	79 (73,8)	0,859
SGLT2i (n,%)	22 (35,5)	39 (36,4)	0,900
Diuréticos de alça (n,%)	33 (52,4)	56 (53,3)	0,813

*SSH: solução salina hipertônica; LD: diurético de alça; FEVE: fração de ejeção do ventrículo esquerdo; DAC: doença arterial coronariana; DRC: doença renal crônica; RASi: inibidores do sistema renina-angiotensina-aldosterona; ARM: antagonistas dos receptores mineralocorticoides; SGLT2i: inibidores do cotransportador 2 de sódio-glicose.*

### Eficácia e parâmetros laboratoriais

A natriurese na 2ª hora (p=0,001) e o débito urinário nas primeiras 24 horas (p=0,001) foram maiores no grupo combo inicial do que no grupo DA padronizado (Tabela 2). Uma redução estatisticamente significativa no peso corporal e na escore de congestão foi observada em ambos os grupos de tratamento (Tabela 3, Tabela Suplementar 2). No grupo DA padronizado, houve elevação significativa dos níveis de Cr (p=0,009) e declínio simultâneo da TFGe (p=0,050) ao final do tratamento. Os níveis de Cr (p=0,218) e TFGe (p=0,616) não foram alterados em pacientes com IC que receberam SSH antecipadamente (Tabela 3). Uma pequena diferença no tamanho do efeito foi observada entre as duas estratégias de tratamento em relação à Cr e TFGe (r = -0,243 para Cr e r = -0,195 para TFGe) (Tabela Suplementar 2). A mediana da alteração do nível sérico de Na+ após a administração de SSH foi de 3 (1-8) mEq/L (p=0,001), enquanto não houve alteração significativa observada nos níveis séricos de Na+ com a administração de DA. Embora a diferença entre os tamanhos de efeito das duas estratégias de tratamento no Na+ sérico tenha sido estatisticamente significativa, verificou-se que houve um tamanho de efeito médio (r=-0,377, p=0,001) (Tabela Suplementar 2). Embora tenha sido observada uma diminuição nos níveis séricos de Cl- com a administração de DA (p=0,001), o Cl- sérico permaneceu constante com a combinação inicial de SSH (p=0,295). Os níveis de BNP diminuíram significativamente entre todos os pacientes, independentemente das estratégias de tratamento diurético empregadas (p=0,001 para a combinação inicial de SSH e DA padronizado), e o declínio observado nos níveis de BNP foi determinado como sendo um efeito semelhante em ambas as abordagens de tratamento (r=- 0,012) (Tabela 3, Tabela 2 suplementar). Além disso, não houve diferença entre os níveis de BNP na admissão e na alta nas duas estratégias de tratamento (Tabela Suplementar 3). A administração de SSH resultou em um aumento nos níveis de Hct durante o tratamento, enquanto os níveis de Hct permaneceram estáveis com DA (p=0,038 para SSH combinado inicial, p = 0,573 para DA padronizado) (Tabela 3).

**Tabela 2:** – Comparação dos parâmetros de eficácia de acordo com duas estratégias de tratamento diurético

	SSH+LD Combinação inicial (n = 63)	LD Padronizado (n = 108)	p
Débito urinário no 1º dia (mL)*	3975,00 (3000,00-5150,00)	2583,33 (2000,00-3250,00)	**0,001**
Na+ urinário na 2ª hora (mEq/L)	116,00 (82,75-126,00)	68,50 (54,00-89,75)	**0,001**

SSH: solução salina hipertônica; LD: diuréticos de alça. *O débito urinário foi definido como a resposta da estratégia diurética durante as primeiras 24 horas.

**Tabela 3:** – Comparação de variáveis laboratoriais, peso corporal e escore de congestão para os pacientes do estudo, tanto na admissão quanto na alta, de acordo com duas estratégias de tratamento diurético

	SSH+LD Combinação inicial (n=63)		p	LD padronizado (n=108)		p
	Admissão	Alta		Admissão	Alta	
Peso corporal (kg)	72,20 (67,30-78,30)	68,00 (62.30-74.00)	**0,001**	72,80 (67,70-82,00)	68,40 (63,32-79,32)	**0,001**
Escore de congestão	5,00 (4.00-6.00)	0,00 (0,00-1,00)	**0,001**	5,00 (3,50-6,00)	0,00 (0,00-0,00)	**0,001**
Cr (mg/dL)	1,20 (0,90-1,70)	1,20 (1,00-1,50)	0,218	1,10 (0,90-1,40)	1,20 (0,90-1,70)	**0,009**
TFG (mL/min/1,73m^2^)	48,50 (29,75-72,50)	50,00 (35,50-63,50)	0,616	56,00 (41,00-71,00)	55,00 (35,00-71,00)	**0,050**
Na+ (mEq/L)	137,00(131,75-140,00)	140,00 (136,00-142,25)	**0,001**	139,00 (137,00-141,00)	139,00 (136,00-140,00)	0,470
K+ (mEq/L)	4,60 (4,20-5,00)	4,30 (3,97-4,62)	**0,001**	4,50 (4,10-5,00)	4,20 (4,00-4,60)	**0,001**
Cl- (mEq/L)	99,00 (94,00-103,25)	99,00 (96,00-103,00)	0,295	102,00 (99,00-106,00)	98,00 (95,00-103,00)	**0,001**
Mg++ (mg/dL)	1,70 (1,60-2,10)	1,90 (1,70-2,20)	**0,017**	1,90 (1,60-2,20)	2,00 (1,70-2,20)	0,128
Ca++ (mg/dL)	8,90 (8,50-9,32)	9,00 (8.60-9.40)	0,494	8,90 (8,50-9,50)	8,90 (8,50-9,30)	0,945
BNP (pg/mL)	898,50 (418,00-1725,00)	538,00 (197,69-795,00)	**0,001**	686,00 (332,32-1499,98)	361,72 (183,00-816,00)	**0,001**
Hb (g/dL)	11,00 (9,50-12,70)	11,70 (10,00-12,60)	**0,016**	11,60 (10,60-12,80)	11,95 (10,37-12,92)	0,495
Htc (%)	33,40 (30,25-39,25)	35,30 (31,20-39,00)	**0,038**	35,80 (33,60-39,90)	36,20 (32,87-40,05)	0,573

*SSH: solução salina hipertônica; LD: diuréticos de alça; BUN: nitrogênio ureico no sangue; Cr: creatinina; TFG: taxa de filtração glomerular; BNP: Peptídeo natriurético tipo B; Hb: hemoglobina; Htc: hematócrito.*

### Parâmetros e resultados de segurança

A piora da função renal ocorreu em 48 (28,4%) dos pacientes com ICA (10 [16,1%] pacientes no grupo de combinação inicial, 38 [35,5%] pacientes no grupo de DA padronizado, p = 0,007). Os pacientes que receberam SSH apresentaram menor tempo de internação hospitalar do que aqueles que receberam DA (4 dias [3-7 dias] vs. 5 dias [4-7 dias], p=0,004). Não houve diferenças na necessidade de hemodiálise, internação por IC, mortalidade hospitalar e mortalidade geral entre as duas abordagens de tratamento (Tabela 4).

**Tabela 4:** – Comparação dos resultados entre duas estratégias de tratamento diurético

	SSH+LD Combinação inicial (n = 63)	LD Padronizado (n = 108)	p
**Resultados**			
WRF (n,%)	10 (16,1)	38 (35,5)	**0,007**
Tempo de internação (dia)	4 (3-7)	5 (4-7)	**0,004**
Necessidade de hemodiálise (n,%)	1 (1,6)	2 (1,9)	0,899
Internação por IC* (n,%)	5 (7,9)	14 (13,0)	0,313
Mortalidade hospitalar (n,%)	2 (3,2)	5 (4,7)	0,649
Mortalidade por todas as causas* (n,%)	3 (4,8)	3 (2,8)	0,496

WRF: piora da função renal; IC: insuficiência cardíaca. *A hospitalização por insuficiência cardíaca e a mortalidade por todas as causas foram avaliadas nos primeiros 6 meses após a hospitalização índice.

## Discussão

Esta análise retrospectiva fornece a eficácia do tratamento inicial da SSH combinada com DA com um perfil de segurança favorável em pacientes com ICA. Não houve deterioração discernível nos níveis séricos de Na+ ou outros níveis de eletrólitos, na função renal e no tempo de internação hospitalar em pacientes com ICA que receberam SSH precoce. Na verdade, a diurese no 1º dia de tratamento e os níveis urinários de Na+ na 2ª hora foram maiores no grupo da combinação inicial. Além disso, não houve diferenças significativas nos desfechos duros entre os pacientes que receberam administração precoce de SSH, exceto PFR.

A administração de SSH via oral ou IV tem potencial para melhorar a função glomerular através da sua capacidade de aumentar o volume plasmático em até 30%. Portanto, a SSH também pode aumentar a perfusão renal, bloquear o sistema renina-angiotensina-aldosterona e induzir vasodilatação nos territórios renais.^8,12,19,20^ Na verdade, pela primeira vez, Issa et al. mostraram que pacientes tratados com SSH+DA na fase aguda apresentaram menor creatinina sérica e cistatina C em comparação aos pacientes tratados apenas com DA.^12^ Em nosso estudo, foi consistentemente observado que a TFGe não diminuiu com o grupo inicial de combinação SSH + DA, em contraste com o único grupo DA. A ausência de diminuição da TFG pode encorajar a utilização de SSH como parte do tratamento diurético inicial para uma diurese mais eficaz durante a hospitalização por ICA.

Uma grande barreira para a adoção generalizada da SSH é a preocupação entre os médicos de que seu uso possa exacerbar a IC devido à alta concentração de Na+ presente na SSH.^7^ No entanto, nenhum dos estudos anteriores mostrou evidências de que a SSH leva a níveis séricos de Na+ significativamente elevados após agravamento da IC ou deterioração neurológica.^7,21-23^ Na verdade, um estudo prospectivo controlado por placebo conduzido por Issa et al. não encontrou diferenças significativas nos níveis de Na+ entre as duas estratégias de tratamento durante a fase aguda.^12^ No entanto, nosso estudo observou um aumento moderado nos níveis de Na+ em pacientes tratados com a combinação inicial de SSH+DA. As principais diferenças neste achado contrastante podem ser atribuídas ao protocolo SSH e ao desenho do estudo. Primeiramente, Issa et al. planejaram o protocolo de SSH como um curso de três dias de 100 mL de SSH duas vezes ao dia, uma infusão de 1 hora e infusão de solução salina foram administradas no grupo placebo.^12^ Em segundo lugar, embora o estudo de Issa et al. estudo foi prospectivo e controlado por placebo,^12^ nosso estudo teve um tamanho amostral maior e as características basais dos pacientes em ambos os grupos de terapia foram iguais. No geral, em contraste com as preocupações dos médicos, os níveis séricos de Na+ ligeiramente aumentados em uma janela de tempo precoce também podem estar associados a um aumento temporário nos níveis séricos basais de peptídeo natriurético atrial (ANP) devido ao reabastecimento plasmático.^24^ Assim, o aumento dos níveis de ANP pode resultar em melhora da diurese por vasodilatação renal, atingindo o peso seco mais rapidamente e em uma redução mais rápida dos níveis de BNP de acordo com a estratégia padronizada de DA. De fato, em nossa coorte de pacientes, descobriu-se que a queda nos níveis de BNP no mesmo nível foi mais rápida na combinação inicial de SSH + DA em comparação com o grupo de regime apenas com DA. Assim, este método terapêutico pode oferecer uma abordagem custo-efetiva para reduzir o tempo de permanência no hospital e minimizar os custos de saúde. A realização de investigações abrangentes e orientadas para os custos em grande escala produzirá uma ilustração mais clara deste conceito.

As respostas sensoriais de sal e volume, incluindo *feedback* tubuloglomerular e liberação de renina, são reguladas principalmente pelo cloreto no rim.^25,26^ Portanto, o cloreto parece ser o principal fator que influencia a capacidade do rim de detectar sobrecarga de volume.^27^ Recentemente, uma família de serina-treonina quinases (sem lisina [K] [WNK]) foi reconhecida como um sensor molecular para sal e um regulador significativo da hemostasia eletrolítica e das funções diuréticas.^27^ O cloreto ativa o cotransportador Na+/K+/2CI- e o cotransportador Na+/Cl- através do sistema WNK. Em última análise, o cloreto serve como um inibidor da reabsorção renal de sal com estes transportadores específicos.^27,28^ A redução nas concentrações de cloreto no plasma leva ao aumento da reabsorção renal de sal, promovendo consequentemente o surgimento de resistência diurética entre os pacientes que recebem diuréticos.^28^ Lamentavelmente, o DA para terapia de congestão resulta na diminuição dos níveis de cloreto e em uma resposta inadequada à administração de DA.^29^ Assim, a administração inicial inicial de SSH durante o tratamento pode prevenir eficazmente o aparecimento de resistência diurética, evitando um declínio nos níveis de Cl-, oferecendo assim uma resposta diurética segura em pacientes com ICA que estão recebendo DA.^30^

Em nossa investigação, observamos um aumento significativo nos níveis de Hct entre os pacientes inicialmente tratados com uma combinação de SSH e DA, em contraste com aqueles tratados apenas com DA. Esta observação está alinhada com a literatura existente, em que a remoção de fluidos através do tratamento diurético na ICAD tem sido associada à hemoconcentração. Além disso, a hemoconcentração tem sido correlacionada com o aumento das doses de diuréticos, maior redução do peso corporal, menor tempo de internação hospitalar e melhor sobrevida.^31,32^ Vale a pena enfatizar que um regime diurético guiado por hemoconcentração pode ser promissor na avaliação do estado ideal de congestão e potencialmente prever resultados clínicos significativos em pacientes com ICAD. A não exibição de um nível de Hct aumentado durante um regime diurético pode ser indicativa de um prognóstico adverso, como PFR e uma internação hospitalar prolongada. Em nosso estudo, os pacientes tratados com SSH+DA apresentaram aumento acentuado nos níveis de Hct. Esse aumento correspondeu a uma diminuição da incidência de PFR e a um menor tempo de internação hospitalar, ressaltando assim os benefícios potenciais das abordagens terapêuticas guiadas por hemoconcentração.

Seguindo os dados da literatura,^33^ o tempo de internação hospitalar foi menor no grupo SSH do que no grupo DA. Esse achado pode ser potencialmente esclarecido pelo maior nível de débito urinário e TFGe estabilizada durante a hospitalização no grupo SSH desde o início da estratégia diurética durante a internação hospitalar.

A piora da função renal tem sido geralmente observada com a administração de DA em pacientes com IC por diversas razões, incluindo vasoconstrição de arteríolas aferentes e ativação do sistema renina-angiotensina-aldosterona.^34,35^ A administração de SSH como tratamento inicial combinado com DA pode ter o potencial de mitigar os fatores acima mencionados que contribuem para a PFR em pacientes com ICA.^20,36^ Assim, o risco de desenvolver PFR pode ser relativamente menor com a administração precoce de SSH. Isto sugere que a administração precoce de SSH poderia servir como uma opção terapêutica segura desde o início da estratégia diurética, particularmente para pacientes que apresentam IC e DRC.

Embora tenha havido resultados conflitantes em termos de mortalidade por todas as causas em pacientes com ICA que receberam SSH, estudos indicaram que um maior tempo de acompanhamento revela uma vantagem de sobrevida associada à SSH em pacientes com ICA.^11,21^ No presente estudo, analisamos a mortalidade por todas as causas nos primeiros 6 meses após a alta hospitalar. A duração limitada do período de acompanhamento pode ter dificultado a determinação das diferenças na mortalidade por todas as causas entre as duas diferentes estratégias de tratamento diurético. Além disso, a mortalidade hospitalar e a hospitalização por IC nos primeiros 6 meses não foram diferentes entre os dois grupos de tratamento, apesar dos escores de congestão semelhantes na alta. A observação desse achado pode ser atribuída às características basais bastante semelhantes dos pacientes.

### Limitações

Existem algumas limitações que vale a pena mencionar. Devido à natureza retrospectiva do estudo, devemos interpretar nossos achados com cautela. Embora o débito urinário das primeiras 24 horas tenha sido calculado, o débito urinário total durante a internação do paciente permaneceu desconhecido. A potencial falta de significância estatística nas diferenças observadas nos desfechos difíceis entre as duas estratégias de tratamento pode ser atribuída ao tamanho limitado da amostra de pacientes incluídos em nosso estudo. Além disso, podemos ter rastreado num período relativamente curto a ocorrência de maus resultados. Estudos com maior tempo de triagem ou acompanhamento podem preencher essa lacuna. Os dados estavam sujeitos a viés de seleção.

## Conclusão

A solução salina hipertônica como parte de uma combinação inicial com DA pode fornecer uma diurese mais segura e eficaz sem prejudicar a função renal, mesmo em pacientes com DRC. A administração precoce de SSH sem esperar pela resposta do DA pode levar a uma menor internação hospitalar e a uma menor taxa de necessidade de hemodiálise em comparação com a administração de DA isoladamente em pacientes com ICA. Portanto, o regime combinado inicial de SSH+DA pode diminuir os custos de saúde na ICA.
