# Supplementary material for: O Efeito da Administração Precoce de Solução Salina Hipertônica na Insuficiência Cardíaca Descompensada Aguda
Source: Arq Bras Cardiol. 2024 Jul 1;121(7):e20230818. [Article in Portuguese] doi: 10.36660/abc.20230818 (PMC12080712; doi:10.36660/abc.20230818)
Supplement: Translation

# Translation: The Effect of Early Administration of Hypertonic Saline Solution İn Acute Decompensated Heart Failure

**DOI:** 10.36660/abc.20230818i

**Authors:** Tugce Colluoglu, Tuğba Kapanşahin, Melahat Hicran Aksu, Orhan Önalan, Yeşim Akin

**Affiliations:** 1 Turkey Karabuk University, Faculty of Medicine, Department of Cardiology, Karabuk – Turkey

**Keywords:** Hypertonic Saline Solution, Kidney Function Tests, Heart Failure

## Abstract

**Background:**

There was no scientific evidence about the initial treatment of hypertonic saline solution (HSS) in acutely decompensated heart failure (ADHF).

**Objectives:**

This study assessed the impact of using HSS along with a loop diuretic (LD) as the first diuretic treatment for ADHF, focusing on renal function, electrolyte levels, and clinical outcomes.

**Methods:**

In this retrospective case-control study, 171 adult patients (93 females/78 males) with ADHF were included between January 1, 2022, and December 31, 2022. Patients were allocated into two groups: upfront combo HSS+LD and standardized LD. The primary endpoint was worsening renal function (WRF). Hospitalization for HF and all-cause mortality were evaluated during 6 months of follow-up. The significance level adopted in the statistical analysis was 5%.

**Results:**

The groups exhibited similarities in baseline characteristics.A significantly higher diuresis on the 1^st^ day (3975 [3000-5150] vs. 2583 [2000-3250], p=0.001) and natriuresis on the 2^nd^ hour (116.00 [82.75-126.00] vs. 68.50 [54.00-89.75], p=0.001) in the initial upfront combo HSS+LD were found in comparison with the standardized LD.When compared to the standardized LD, the utilization of HSS led to an increase in serum Na^+^ (137.00 [131.75-140.00] vs. 140.00 [136.00-142.25], p=0.001 for upfront combo HSS, 139.00 [137.00-141.00] vs. 139.00 [136.00-140.00], p=.0470 for standardized LD), while chloride (99.00 [94.00-103.25] vs. 99.00[96.00-103.00], p=0.295), GFR (48.50 [29.75-72.50 vs. 50.00 [35.50-63.50, p=0.616), and creatinine (1.20 [0.90-1.70] vs. 1.20 [1.00-1.50], p=0.218) remained stable in the upfront combo HSS group when compared to standardized LD group (Cl^-^: 102.00 [99.00-106.00] vs. 98.00 [95.00-103.00], p=0.001, eGFR: 56.00 [41.00-71.00] vs. 55.00 [35.00-71.00], p=0.050, creatinine:1.10 [0.90-1.40] vs. 1.20 [0.90-1.70], p=0.009). Worsening renal function (16.1% vs 35.5%, p=0.007), and length of stay in the hospital (4 days [3-7] vs. 5 days [4-7], p=0.004) were lower in the upfront combo HSS+LD in comparison with the standardized LD. In-hospital mortality, hospitalization for HF, and all-cause mortality were similar between the two groups.

**Conclusion:**

HSS as an initial therapy, when combined with LD, may provide a safe and effective diuresis without impairing renal function in ADHF. Therefore, HSS may lead to a shorter length of stay in the hospital for these patients.

## Introduction

Worsening heart failure (WHF) is associated with increased mortality and morbidity, contributing a significant economic burden on society. These episodes are the leading factor behind hospitalization for heart failure (HF), mainly due to worsening congestion requiring intravenous (IV) diuretics.^1^ Current HF guidelines recommend a staged diuretic regimen which includes the administration of IV loop diuretics as a first-line therapy for worsening congestion. However, with IV loop diuretics alone, the time needed to achieve optimal decongestion is extended, resulting in many patients with HF being discharged with residual congestion. The presence of residual congestion is linked to unfavorable outcomes and early readmission.^2,3^ Of note, proximal tubule diuretics are being tested to improve decongestive therapy on top of standard loop diuretic-based treatment including acetazolamide, and sodium-glucose cotransporter 2 inhibitors.^3-6^

In the literature, oral NaCI or IV 3% NaCI-hypertonic saline solution (HSS) has been used to improve diuretic response in refractory, acutely decompensated HF patients with a safe profile. Urine output and weight loss were significantly increased with the administration of HSS and high-dose IV loop diuretic in this population.^7-9^ However, the above-mentioned studies evaluated the effect of HSS as a bail-out option among patients with refractory, acutely decompensated HF.^7,9-11^ The effect of the initial administration of HSS as a part of an upfront diuretic regimen has been poorly studied in acutely decompensated HF.^12^ As we examine the existing body of literature, it becomes evident that there is a research gap in investigating the potential benefit of proactive administration of HSS as opposed to its conventional use as a rescue intervention, particularly following the publication of significant trials that have linked its use with a combined diuretic regimen as an initial therapy in ADHF. Therefore, we aim to evaluate whether the initial addition of HSS to standardized IV loop diuretic therapy among patients with acutely decompensated HF would improve diuretic response, successful decongestion, length of hospital stay, and hard endpoints safely.

### Study design and study population

This study was conducted as a retrospective case-control study. The study protocol was approved and conducted following the Karabuk University Ethics Committee for Non-Interventional Clinical Research’s approval number 2023/1294. The STROBE guidelines for case-control studies and a checklist were used in the preparation of this manuscript.^13^ The study was performed in accordance with the principles stated in the Declaration of Helsinki.

### Sample size calculation

According to the Kelsey Formula, 111 (43/68) patients are required for an 80% power, and we determined a 70% relative risk reduction in primary outcome with HSS therapy based on Issa et al.’s trial.^12,14^

### Data collection

We identified all patients across the entire range of left ventricular ejection fraction (LVEF) hospitalized with various cardiac causes between January 1, 2022, and December 31, 2022, in the coronary intensive care unit at Karabuk University Hospital from the hospital’s electronic medical records (n=1263). Inclusion criteria were evidence of acutely decompensated HF, which was confirmed by the elevated levels of B-type natriuretic peptide (BNP) (≥100 pg/mL), congestion findings in echocardiography, and chest X-ray or thorax computed tomography. Patients receiving HSS as a bailout treatment who did not receive it on the first day (n=38), patients receiving IV loop diuretics as an out of defined study protocol (n=30), estimated glomerular filtration rate (eGFR) ≤20 mL/min/1.73m^2^(n=102), systolic blood pressure ≤90 mmHg (n=43) or signs of hypoperfusion defined by the levels of lactate of ≥2mmol/L (n=24), hypernatremia (Na^+^ ≥145mEq/L) (n=44), acute pulmonary edema (n=121), acute coronary syndrome (n=455), arrhythmic events (n=59), and primary valvular disease (n=176) were not included. Hence, this study included 171 patients with acutely decompensated HF. The control group consisted of 108 consecutively chosen age- and sex-matched patients whose diuretics were administered as IV bolus loop diuretics only (Figure 1).

**Figure 1:** – Flow chart. &: In cases of inadequate response to iv.loop diuretics, the dose of iv.loop diuretics is increased twofold. * Hospitalization for heart failure and all-cause mortality were evaluated in the first 6 months after index hospitalization. eGFR: estimated glomerular filtration rate; IV: intravenous; HSS: hypertonic salin solution; AHF: acutely decompensated heart failure; LD: loop diuretic; BUN: blood urine nitrogen; Cr: creatinine; BNP: brain natriuretic peptide; Htc: hematocrit.

Variables such as age, sex, comorbidities, prescribed guideline-directed medical therapy for heart failure, prescribed loop diuretics, date of hospitalization, length of stay in the hospital, hemodialysis status, and date of death were reported. Laboratory variables, including renal function tests (blood urine nitrogen, creatinine [Cr], eGFR), BNP, electrolyte status (Na^+^, K^+^, Cl^-^, Mg^++^, Ca^++^), urinary Na^+^ on the 2^nd^hour, hemoglobin, and hematocrit (Htc), were obtained from the hospital’s electronic record database. Body weight and daily urine output were obtained from the daily physical examination report. Chronic kidney disease (CKD) was defined as an estimated glomerular filtration rate (eGFR) of <60 mL/min/1.73m^2^calculated for at least 3 months.^15^ Length of stay in the hospital was obtained from the hospital’s electronic healthcare database. Data on deaths was obtained from the Turkish Death Notification System.

### Diuretic and hypertonic saline solution protocol

Intravenous loop diuretics were administered at twice the dose of home diuretics according to the 2022 AHA/ACC/HFSA Guideline for the Management of Heart Failure.^16^ The case group consisted of 63 patients who were administered 150 mL of HSS over 30 minutes (300 mL/h) twice a day from the first day of hospitalization until discharge.^7^

### The measurement of left ventricular ejection fraction

All echocardiographic examinations were performed with the Philips EPIQ-7C Ultrasound System for Cardiology (Andover, USA) with an X5-1 probe both on admission and at discharge. All echocardiographic data were stored for offline analysis. LVEF was measured using the modified biplane Simpson’s method.

### The definition of outcomes and safety parameters

The primary endpoint was worsening renal function, which was defined as an increase in plasma creatinine level of ≥0.3 mg/dL during the diuretic treatment strategy.^17^ Secondary endpoints were the need for hemodialysis, successful decongestion, length of stay in the hospital, hospitalization for HF, and all-cause mortality. Urine output was defined as the response to the diuretic strategy during the first 24 hours. Congestion was assessed twice: at the time of admission and at the time of discharge from the hospital. We conducted a comprehensive evaluation to assess both pulmonary and systemic congestion. The congestion score was established through the use of physician documentation and a simple score system that assigned one point for each congestion finding (Supplementary Table 1). Length of hospital stay was defined as the period between the date of hospital admission and the date of hospital discharge. Hospitalization for HF and all-cause mortality were evaluated in the first 6 months after the date of index discharge.

### Statistical analysis

The normality of continuous variables was evaluated through the Kolmogorov-Smirnov test. Continuous variables were expressed as mean ± standard deviation, or median (interquartile range) as appropriate. Categorical variables were expressed as numbers and percentages. The Chi-Square test, for categorical variables, and unpaired Student t-test or Mann-Whitney U test, for continuous variables, were performed to compare baseline characteristics and treatment effects between two treatment strategies, as appropriate. To assess the differences in laboratory parameters and body weight before and after two distinct treatment strategies, we performed the Wilcoxon test. The effect size between the two treatment strategies was analyzed by Cohen’s r effect size: z/√n. The definition of effect size, as calculated by the r coefficient, is: r=0.10 is considered a small effect, r=0.30 is a medium effect, and r=0.50 is a large effect.^18^ A two-tailed p-value of 0.05 was considered statistically significant. All analyses were conducted using SPSS statistical software version 25.0.

## Results

### Baseline characteristics

The baseline characteristics of 171 patients are summarized in Table 1. The mean age of patients was 73.7±10.1 years, and 93 (54%) of the patients were women. Patients with HF were classified into two groups based on the initial diuretic treatment approach (Table 1).

**Table 1:** – Baseline characteristics of the study population

	Upfront combo HSS+LD (n = 63)	Standardized LD (n = 108)	p
**Age (years)**	74.3± 9.2	73.3±10.7	0.557
**Sex (F/M)**	36/27	57/51	0.580
**LVEF (%)**	45.00 (35.00-55.00)	45.00 (35.00-59.00)	0.414
**Comorbidities**			
Hypertension (n,%)	50 (79.4)	92 (85.2)	0.328
Diabetes Mellitus (n,%)	35 (55.6)	57 (52.8)	0.725
CAD (n,%)	27 (42.9)	52 (48.1)	0.503
Hyperlipidemia (n,%)	25 (39.7)	46 (42.6)	0.710
CKD (n,%)	45 (76.3)	77 (68.8)	0.301
Atrial Fibrillation (n,%)	31 (50.0)	50 (46.7)	0.682
**Baseline Medical Therapy**			
Beta blockers (n,%)	60 (96.8)	101 (94.4)	0.482
RASi (n,%)	40 (64.5)	79 (73.8)	0.201
MRA (n,%)	45 (72.6)	79 (73.8)	0.859
SGLT2i (n,%)	22 (35.5)	39 (36.4)	0.900
Loop diuretics (n,%)	33 (52.4)	56 (53.3)	0.813

*HSS: hypertonic saline solution; LD: loop diuretic; LVEF: left ventricular ejection fraction; CAD: coronary artery disease; CKD: chronic kidney disease; RASi: renin-angiotensin-aldosterone system inhibitors; MRA: mineralocorticoid receptor antagonists; SGLT2i: sodium-glucose co-transporter 2 inhibitors.*

### Efficacy and laboratory parameters

Natriuresis in the 2^nd^hour (p=0.001) and urinary output in the first 24 hours (p=0.001) were greater in the upfront combo group than in the standardized LD group (Table 2). A statistically significant reduction in body weight and congestion score was observed in both treatment groups (Table 3, Supplementary Table 2). In the standardized LD group, there was a significant elevation of Cr levels (p=0.009) and a simultaneous decline in eGFR (p=0.050) at the end of the treatment. The levels of Cr (p=0.218) and eGFR (p=0.616) were not changed in patients with HF receiving upfront HSS (Table 3). A small difference in effect size was noted between the two treatment strategies in relation to Cr and eGFR (r=-0.243 for Cr and r=-0.195 for eGFR) (Supplementary Table 2). The median serum Na^+^ level change after the administration of HSS was 3 (1-8) mEq/L (p=0.001), while there was no significant change observed in serum Na^+^ levels with the administration of LD. Although the difference between the effect sizes of the two treatment strategies on serum Na^+^ was statistically significant, it was found to have a medium effect size (r=-0.377, p=0.001) (Supplementary Table 2). While a decrease in serum Cl^-^levels was observed with the administration of LD (p=0.001), serum Cl^-^ remained constant with the upfront HSS combo (p=0.295). BNP levels decreased significantly among all patients, irrespective of the diuretic treatment strategies employed (p=0.001 for upfront combo HSS and standardized LD), and the observed decline in BNP levels was determined to be a similar effect in both treatment approaches (r=-0.012) (Table 3, supplementary Table 2). Additionally, there was no difference between BNP levels on admission and at discharge in the two treatment strategies (Supplementary Table 3). The administration of HSS resulted in an increase in Hct levels during the treatment, while Hct levels remained stable with LD (p=0.038 for upfront combo HSS, p=0.573 for standardized LD) (Table 3).

**Table 2:** – The comparison of efficacy parameters according to two diuretic treatment strategies

	Upfront combo HSS+LD (n = 63)	Standardized LD (n = 108)	p
Urine output on the 1st day (mL)*	3975.00 (3000.00-5150.00)	2583.33 (2000.00-3250.00)	**0.001**
Urinary Na+ on the 2nd hour (mEq/L)	116.00 (82.75-126.00)	68.50 (54.00-89.75)	**0.001**

HSS, hypertonic saline solution; LD, loop diuretics. *Urine output was defined as the response of the diuretic strategy during the first 24 hours.

**Table 3:** – The comparison of laboratory variables, body weight, and congestion score for study patients both on admission and at discharge according to two diuretic treatment strategies

	Upfront combo HSS+LD (n = 63)		p	Standardized LD (n = 108)		p
	Admission	Discharge		Admission	Discharge	
Body weight (kg)	72.20 (67.30-78.30)	68.00 (62.30-74.00)	**0.001**	72.80 (67.70-82.00)	68.40 (63.32-79.32)	**0.001**
Congestion score	5.00 (4.00-6.00)	0.00 (0.00-1.00)	**0.001**	5.00 (3.50-6.00)	0.00 (0.00-0.00)	**0.001**
Cr (mg/dL)	1.20 (0.90-1.70)	1.20 (1.00-1.50)	0.218	1.10 (0.90-1.40)	1.20 (0.90-1.70)	**0.009**
GFR (mL/min/1.73m2)	48.50 (29.75-72.50)	50.00 (35.50-63.50)	0.616	56.00 (41.00-71.00)	55.00 (35.00-71.00)	**0.050**
Na+ (mEq/L)	137.00(131.75-140.00)	140.00 (136.00-142.25)	**0.001**	139.00 (137.00-141.00)	139.00 (136.00-140.00)	0.470
K+ (mEq/L)	4.60 (4.20-5.00)	4.30 (3.97-4.62)	**0.001**	4.50 (4.10-5.00)	4.20 (4.00-4.60)	**0.001**
Cl- (mEq/L)	99.00 (94.00-103.25)	99.00 (96.00-103.00)	0.295	102.00 (99.00-106.00)	98.00 (95.00-103.00)	**0.001**
Mg++ (mg/dL)	1.70 (1.60-2.10)	1.90 (1.70-2.20)	**0.017**	1.90 (1.60-2.20)	2.00 (1.70-2.20)	0.128
Ca++ (mg/dL)	8.90 (8.50-9.32)	9.00 (8.60-9.40)	0.494	8.90 (8.50-9.50)	8.90 (8.50-9.30)	0.945
BNP (pg/mL)	898.50 (418.00-1725.00)	538.00 (197.69-795.00)	**0.001**	686.00 (332.32-1499.98)	361.72 (183.00-816.00)	**0.001**
Hb (g/dL)	11.00 (9.50-12.70)	11.70 (10.00-12.60)	**0.016**	11.60 (10.60-12.80)	11.95 (10.37-12.92)	0.495
Htc (%)	33.40 (30.25-39.25)	35.30 (31.20-39.00)	**0.038**	35.80 (33.60-39.90)	36.20 (32.87-40.05)	0.573

*HSS: hypertonic saline solution; LD: loop diuretics; BUN, blood urea nitrogen; Cr: creatinine; GFR: glomerular filtration rate; BNP: B-type natriuretic peptide; Hb: hemoglobin; Htc: hematocrit.*

### Safety parameters and outcomes

Worsening renal function occurred in 48 (28.4%) of patients with AHF (10 [16.1%] patients in the upfront combo group, 38 [35.5%] patients in the standardized LD group, p=0.007). Patients receiving HSS exhibited a shorter length of stay in the hospital than those receiving LD (4 days [3-7 days] vs. 5 days [4-7 days], p=0.004). There were no differences in the necessity for hemodialysis, hospitalization for HF, in-hospital mortality, and overall mortality between the two treatment approaches (Table 4).

**Table 4:** – The comparison of outcomes between two diuretic treatment strategies

	Upfront combo HSS+LD (n = 63)	Standardized LD (n = 108)	p
**Outcomes**			
WRF (n,%)	10 (16.1)	38 (35.5)	**0.007**
Length of hospital stay (day)	4 (3-7)	5 (4-7)	**0.004**
Need for hemodialysis (n, %)	1 (1.6)	2 (1.9)	0.899
Hospitalization for HF* (n, %)	5 (7.9)	14 (13.0)	0.313
In-hospital mortality (n, %)	2 (3.2)	5 (4.7)	0.649
All-cause mortality* (n, %)	3 (4.8)	3 (2.8)	0.496

WRF, worsening renal function; HF, heart failure. *Hospitalization for heart failure and all-cause mortality were evaluated in the first 6 months after index hospitalization.

## Discussion

This retrospective analysis provides the efficacy of the initial treatment of HSS combined with LD with a favorable safety profile in patients with AHF. There was no discernible deterioration in serum Na^+^ levels or other electrolyte levels, renal function, and length of stay in the hospital in patients with AHF who received early HSS. In fact, diuresis on the 1^st^day of treatment and urinary Na^+^ levels on the 2^nd^ hour were found to be higher in the upfront combo group. Additionally, there were no significant differences in hard endpoints among patients who received early administration of HSS, except for WRF.

The administration of oral or IV HSS has the potential to improve glomerular function through its ability to increase plasma volume by up to 30%. Therefore, HSS can also increase renal perfusion, block the renin-angiotensin-aldosterone system, and induce vasodilatation in renal territories.^8,12,19,20^ Indeed, for the first time, Issa et al. showed that patients treated with HSS+LD in the acute phase exhibited lower serum creatinine and cystatin C compared to the patients treated with only LD.^12^ In our study, it was consistently observed that eGFR did not decrease with the initial upfront combo HSS+LD group in contrast to the only LD group. The absence of a decrease in GFR may encourage the utilization of HSS as a part of initial diuretic treatment for more effective diuresis during hospitalization for AHF.

A large barrier to the widespread adoption of HSS is the concern among physicians that its usage may exacerbate HF due to the high Na^+^ concentration present in HSS.^7^ However, none of the previous studies has shown evidence that HSS leads to significantly elevated serum Na^+^ levels following worsening HF or neurological deterioration.^7,21-23^ In fact, a prospective, placebo-controlled study conducted by Issa et al. found no significant differences in Na^+^ levels between the two treatment strategies during the acute phase.^12^ However, our study observed a moderate increase in Na^+^ levels in patients treated with upfront combo HSS+LD. The main differences in this contrasting finding may be attributed to the HSS protocol and study design. Firstly, Issa et al. planned the HSS protocol as a three-day course of 100 mL of HSS twice a day, a 1-hour infusion and, saline infusion were given in the placebo group.^12^ Secondly, although the Issa et al. study was prospective and placebo-controlled,^12^ our study had a larger sample size, and the baseline characteristics of patients in both therapy groups were equal. Overall, in contrast to physicians’ concerns, slightly increased serum Na^+^ levels at an early time window may also be associated with a temporary increase in baseline serum atrial natriuretic peptide (ANP) levels due to plasma refilling.^24^ Thus, increased ANP levels can result in improved diuresis by renal vasodilatation, reaching dry weight more rapidly, and a faster reduction in BNP levels according to standardized LD strategy. Indeed, in our patient cohort, it emerged that the drop in BNP levels at the same level was more rapid in the initial upfront combo HSS+LD compared to the LD-only regimen group. Thus, this therapy method may offer a cost-effective approach to reducing the length of stay in the hospital and minimizing healthcare costs. Conducting comprehensive, cost-oriented investigations on a large scale will yield a more clear illustration of this concept.

Salt and volume sensory responses, including tubuloglomerular feedback and renin release, are primarily regulated by chloride in the kidney.^25,26^ Therefore, chloride appears to be the primary factor that influences the kidney’s capacity to detect volume overload.^27^ Recently, a family of serine-threonine kinases (with-no-lysine [K] [WNK]) has been recognized as a molecular sensor for salt and a significant regulator of electrolyte hemostasis and diuretic functions.^27^ Chloride activates the Na^+^/K^+^/2CI^-^cotransporter and the Na^+^/Cl^-^cotransporter through the WNK system. Ultimately, chloride serves as an inhibitor of renal salt reabsorption with these specific transporters.^27,28^ The reduction in chloride concentrations in plasma leads to increased renal salt reabsorption, consequently promoting the emergence of diuretic resistance among patients receiving diuretics.^28^ Regrettably, LD for congestion therapy results in decreased chloride levels and an inadequate response upon LD administration.^29^ Thus, initial upfront administration of HSS during treatment may effectively prevent the onset of diuretic resistance by averting a decline in Cl^-^ levels, thereby offering a secure diuretic response in patients with AHF who are receiving LD.^30^

In our investigation, we observed a significant rise in Hct levels among patients initially treated with a combination of HSS and LD, in contrast to those treated solely with LD. This observation aligns with existing literature wherein fluid removal through diuretic treatment in ADHF has been linked to hemoconcentration. Additionally, hemoconcentration has been correlated with increased diuretic doses, a greater reduction in body weight, a shorter length of stay in the hospital, and improved survival.^31,32^ It is worth emphasizing that a hemoconcentration-guided diuretic regimen can hold promise in assessing optimal congestion status and potentially predicting significant clinical outcomes in patients with ADHF. Failure to exhibit an increased Hct level during a diuretic regimen might be indicative of an adverse prognosis such as WRF and a prolonged hospital stay. In our study, the patients treated with HSS+LD displayed a marked increase in Hct levels. This increase corresponded to a decreased incidence of WRF and a shorter length of stay in the hospital, thereby underscoring the potential benefits of hemoconcentration-guided therapeutic approaches.

Following the data in the literature,^33^ the length of stay in the hospital was shorter in the HSS group than in the LD group. This finding can be potentially clarified by the higher level of urine output and stabilized eGFR during hospitalization in the HSS group from the start of the diuretic strategy during their hospital stay.

Worsening renal function has generally been observed with the administration of LD in patients with HF for several reasons, including vasoconstriction of afferent arterioles and activation of the renin-angiotensin-aldosterone system.^34,35^ The administration of HSS as an initial treatment combined with LD may have the potential to mitigate the aforementioned factors that contribute to WRF in patients with AHF.^20,36^ Thus, the risk of developing WRF may be relatively lower with the administration of early HSS. It suggests that early administration of HSS could serve as a safe therapeutic option from the beginning of the diuretic strategy, particularly for patients presenting with both HF and CKD.

Although there were conflicting results in terms of all-cause mortality in patients with AHF who received HSS, studies have indicated that an increased follow-up time reveals a survival advantage associated with HSS in patients with AHF.^11,21^ In the present study, we analyzed all-cause mortality within the first 6 months after hospital discharge. The limited duration of the follow-up period may have hindered the determination of differences in all-cause mortality between the two different diuretic treatment strategies. Furthermore, in-hospital mortality and hospitalization for HF in the first 6 months were not different between the two treatment groups despite similar congestion scores at discharge. The observation of this finding can be attributed to the quite similar baseline characteristics of patients.

### Limitations

There are some limitations worth mentioning. Due to the retrospective nature of the study, one should interpret our findings with caution. While the urine output for the first 24 hours was calculated, the total urine output during the patient’s hospital stay remained unknown. The potential lack of statistical significance in the observed differences in hard endpoints between the two treatment strategies could be attributed to the limited sample size of patients included in our study. Moreover, we may have screened in a relatively short period for the occurrence of poor outcomes. Studies with longer screening or follow-up time may fill this gap. The data were subject to selection bias.

## Conclusion

Hypertonic saline solution as a part of an initial upfront combo with LD may provide a safer and more effective diuresis without impairing renal function, even in patients with CKD. Early administration of HSS without waiting for the response of LD may lead to a shorter hospital stay and a lower rate of hemodialysis need in comparison to the administration of LD alone in patients with AHF. Therefore, the upfront combo HSS+LD regimen may decrease healthcare costs in AHF.
